# Health Status of Older US Workers and Nonworkers, National Health Interview Survey, 1997–2011

**DOI:** 10.5888/pcd12.150040

**Published:** 2015-09-24

**Authors:** Diana Kachan, Lora E. Fleming, Sharon Christ, Peter Muennig, Guillermo Prado, Stacey L. Tannenbaum, Xuan Yang, Alberto J. Caban-Martinez, David J. Lee

**Affiliations:** Author Affiliations: Lora E. Fleming, Department of Public Health Sciences, University of Miami Miller School of Medicine, Miami, Florida, European Centre for Environment and Human Health, University of Exeter Medical School, Truro, Cornwall, United Kingdom; Sharon Christ, Department of Human Development and Family Studies and Statistics, Purdue University, West Lafayette, Indiana; Peter Muennig, Mailman School of Public Health, Columbia University, New York, New York; Guillermo Prado, Xuan Yang, Alberto J. Caban-Martinez, David J. Lee, Department of Public Health Sciences, University of Miami Miller School of Medicine, Miami, Florida; Stacey L. Tannenbaum, Sylvester Comprehensive Cancer Center, University of Miami Miller School of Medicine, Miami, Florida.

## Abstract

**Introduction:**

Many US workers are increasingly delaying retirement from work, which may be leading to an increase in chronic disease at the workplace. We examined the association of older adults’ health status with their employment/occupation and other characteristics.

**Methods:**

National Health Interview Survey data from 1997 through 2011 were pooled for adults aged 65 or older (n = 83,338; mean age, 74.6 y). Multivariable logistic regression modeling was used to estimate the association of socioeconomic factors and health behaviors with 4 health status measures: 1) self-rated health (fair/poor vs good/very good/excellent); 2) multimorbidity (≤1 vs ≥2 chronic conditions); 3) multiple functional limitations (≤1 vs ≥2); and 4) Health and Activities Limitation Index (HALex) (below vs above 20th percentile). Analyses were stratified by sex and age (young–old vs old–old) where interactions with occupation were significant.

**Results:**

Employed older adults had better health outcomes than unemployed older adults. Physically demanding occupations had the lowest risk of poor health outcomes, suggesting a stronger healthy worker effect: service workers were at lowest risk of multiple functional limitations (odds ratio [OR], 0.82; 95% confidence interval [CI], 0.71–0.95); and blue-collar workers were at lowest risk of multimorbidity (OR, 0.84; 95% CI, 0.74–0.97) and multiple functional limitation (OR, 0.84; 95% CI, 0.72–0.98). Hispanics were more likely than non-Hispanic whites to report fair/poor health (OR, 1.62; 95% CI, 1.52–1.73) and lowest HALex quintile (OR, 1.21; 95% CI, 1.13–1.30); however, they were less likely to report multimorbidity (OR, 0.78; 95% CI, 0.73–0.83) or multiple functional limitations (OR, 0.82; 95% CI, 0.77–0.88).

**Conclusion:**

A strong association exists between employment and health status in older adults beyond what can be explained by socioeconomic factors (eg, education, income) or health behaviors (eg, smoking). Disability accommodations in the workplace could encourage employment among older adults with limitations.

## Introduction

Adults aged 65 or older are a rapidly expanding segment of the US population, and they are projected to make up approximately 22% of the US workforce by 2022 (1). This population group is becoming increasingly diverse, with a growing proportion of racial/ethnic minorities and women, and the increased rates of workforce engagement in this population can be associated with various health outcomes (2). Older workers are a valuable addition to the workplace because they are on average just as productive as, are more careful and emotionally stable than, and have lower rates of absenteeism than their younger counterparts (3). There is, however, a lack of nationally representative studies comparing the effects of various sociodemographic, health behavior, and occupational factors on the health status of older workers and nonworkers.

Health status varies greatly across sociodemographic groups of older adults. Employed older adults tend to be healthier, both mentally and physically, than their nonworking peers (4,5). Health-related quality of life (HRQL) scores vary across income, education levels, sex, and racial/ethnic groups (6). Blacks are more likely than whites to report functional limitations even after adjustment for age and sex (7), while women, racial/ethnic minorities, and individuals of low socioeconomic status are more likely to report having a disability (8,9).

Overall, studies using nationally representative data have found that 26.0% to 33.2% of adults aged 65 or older report being in fair/poor health, compared with 7.3% to 14.4% of adults younger than 55 (10), and that approximately 27.9% to 48.3% of adults aged 65 or older report great difficulty in at least one of the activities assessing daily function (11). Estimates of multimorbidity prevalence in this population have ranged from 47% to 73% (12). However, these studies had major limitations, including reporting the prevalence of health outcomes without controlling for potential confounders and using data not representative of the entire US population. As a result, these studies may have led to incomplete conclusions. The objective of our study was to characterize 4 major health status measures of older US workers and nonworkers using a representative sample of the US population and controlling for potential sociodemographic and health behavior confounders such as education, race/ethnicity, sex, age, smoking status, alcohol consumption, obesity, and marital status. The results of this study will allow identification of disparities in the heterogeneous aging population to assist in assessing workplace health-related needs and limitations of older adults.

## Methods

### Sample

The National Health Interview Survey (NHIS) is an annual multistage probability household survey of the US civilian noninstitutionalized population. It uses face-to-face interviews to obtain information on sociodemographic and health characteristics. This study used a sample of all adults aged 65 or older (n = 83,338; representing approximately 33,546,235 individuals) from 15 years of NHIS cross-sectional data pooled from 1997 through 2011. Sampling weights were used to ensure unbiased estimates of the national population (13). This study was approved by the University of Miami institutional review board.

### Variables

The 4 main outcomes examined in this study were self-rated health, multimorbidity, functional limitations, and the Health and Activities Limitation Index (HALex, a measure of HRQL). The predictors in all models were employment/occupation, education, race/ethnicity, sex, age, smoking status, and alcohol consumption. Analyses were adjusted for the survey year (as a continuous variable) to control for potential changes in prevalence of the outcomes, as well as employment rates, during the years of the study. Outcome variables were dichotomized for ease of comparison with previous studies.

**Self-rated health. **Participants rated their perceived health on a 5-point Likert scale: 1) excellent, 2) very good, 3) good, 4) fair, 5) poor. These were dichotomized as fair/poor and good or better; this 2-category variable yielded results similar to the multiple-category variable (14).

**Multimorbidity.** Multimorbidity was defined as previous lifetime diagnosis of 2 or more of the following conditions: hypertension, heart disease (including coronary heart disease, angina, and myocardial infarction), stroke, emphysema, asthma, cancer, and diabetes (15).

**Multiple functional limitations.** The number of functional limitations was assessed with the following question: “By yourself, and without using any special equipment, how difficult is it for you to [walk, climb stairs, stand, sit, stoop or kneel, reach over head, grasp, carry heavy objects, push large objects, shop, be social, or relax]?” Participants rated their ability to perform these activities on a 5-point scale from 1 (not at all difficult) to 5 (can’t do at all). A functional limitation was defined as having any difficulty with any one of the above activities. The presence of multiple functional limitations was defined as having a limitation in 2 or more of these activities.

**HALex.** HALex was calculated as described by Livingston and Ko (16). HALex is a utility score that combines information about an individual’s perceived health and activity limitations; the score ranges from 0 to 1, with 0 representing a health state equivalent to death and 1 representing perfect health. The activity limitations included in the index are the following: needing help with personal care or routine needs, having difficulty working, being limited in the kind and amount of work, or being limited in any other way due to health reasons. Participants were dichotomized into those below and those above the 20th percentile value for the population represented (ie, HALex ≤ 0.48 and HALex > 0.48). The 20th percentile was chosen as the cutoff value to model the lowest HALex scores while allowing for sufficient sample size in all population subgroups examined.

### Predictor variables

The primary predictor variable in this study was an employment/occupation hybrid variable. The employment/occupation variable combined information about whether the individual had worked in the week before the NHIS interview (full-time or part-time) and the type of work they reported doing. Type of work was classified as 1) unemployed/retired, 2) white collar worker [reference], 3) service worker, 4) farm worker, and 5) blue collar worker (17). No distinction was made between temporarily unemployed and retired individuals, and both full-time and part-time workers were classified as employed.

The effect of the following variables on the outcomes was examined as well: education (<high school [reference], high school or equivalent, >high school), race/ethnicity (non-Hispanic white [reference], non-Hispanic black, Hispanic, other), sex (male, female [reference]), age (continuous or dichotomized as 65–75 [young–old] and ≥76 [old–old]), smoking history (current, former, never [reference]), alcohol consumption history (current heavy, current light, former, never [reference]), body mass index (BMI < 18.5 or underweight, 18.5 ≤ BMI < 25.0 or normal weight [reference], 25.0 ≤ BMI < 30.0 or overweight, and 30.0 ≤ BMI or obese), and self-identified marital status (married [reference], not married but living with partner, never married, widowed, divorced/separated).

### Statistical analysis

Multiple logistic regression analyses were used to test the associations between employment/occupation and the health outcomes controlling for covariates. Data management and analyses were performed using SAS version 9.3 (SAS Institute Inc). Sample adult NHIS file sampling weights were used to correct for the unequal selection design of the NHIS. In addition, standard errors were adjusted for the nesting of individuals within sampling clusters. Models were tested for the possible interactions of occupation with sex and age to control for the potential differential effects of employment/occupation on the health outcomes for male and female older workers, as well as for age subgroups among older adults. Age was treated as a continuous variable for interaction testing, and it was dichotomized for stratification purposes where such interaction was significant.

## Results

The population had a mean age of 74.6 years (Table 1). More than half (57.2%) were women, and most were non-Hispanic white (82.5%), unemployed/retired (87.1%), and reported no history of heavy drinking in the previous year (95.6%). Approximately two-thirds of those employed worked in white collar professions (7.9%), and only a small fraction (0.3%) were farm workers. Approximately half (50.4%) reported never smoking, and 9.6% were current smokers. Women were less likely than men to report poor health behaviors: 62.1% never smoked and only 1.6% reported heavy drinking, whereas 34.9% of men never smoked and 8.2% of men reported heavy drinking. Women were also less likely than men to be educated beyond high school (35.4% vs 44.6%), and more likely to not work (89.9% vs 83.3%).

**Table 1:** Sample Characteristics of Participants Aged 65 or Older, National Health Interview Survey, 1997–2011^a^

Characteristic	All	Men	Women
**Total**	83,338 (100)	31,988 (42.8)	51,350 (57.2)
**Education**			
Less than high school	25,979 (27.5)	9,688 (26.6)	16,291 (28.2)
High school or equivalent	26,710 (33.2)	8,965 (28.9)	17,745 (36.4)
More than high school	30,649 (39.3)	13,335 (44.6)	17,314 (35.4)
**Race/ethnicity**			
Hispanic	7,778 (6.3)	3,048 (6.3)	4,730 (6.3)
Non-Hispanic white	63,025 (82.5)	24,283 (83.0)	38,742 (82.0)
Black	9,933 (8.2)	3,572 (7.4)	6,361 (8.7)
Other	2,602 (3.1)	1,085 (3.3)	1,517 (2.9)
**Smoking status**			
Never	43,331 (50.4)	11,121 (34.9)	32,210 (62.1)
Former	31,554 (40.0)	17,170 (54.9)	14,384 (28.9)
Current	8,453 (9.6)	3,697 (10.2)	4,756 (9.1)
**Drinking status**			
Never	27,059 (30.1)	5,590 (16.9)	21,469 (40.0)
Former	21,905 (25.9)	9,896 (29.7)	12,009 (23.0)
Current light	30,983 (39.6)	13,861 (45.2)	17,122 (35.4)
Current heavy	3,391 (4.4)	2,641 (8.2)	750 (1.6)
**Marital status**			
Married	34,368 (56.0)	19,321 (73.6)	15,047 (42.7)
Living with partner	792 (1.3)	474 (1.8)	318 (0.9)
Never married	4,198 (3.6)	1,758 (3.5)	2,440 (3.7)
Widowed	33,406 (30.3)	6,405 (13.3)	27,001 (43.0)
Divorced/separated	10,414 (8.9)	3,975 (7.8)	6,439 (9.7)
**Employment/occupation**			
Unemployed/retired	73,138 (87.1)	26,942 (83.3)	46,196 (89.9)
White collar	6,115 (7.9)	2,693 (9.3)	3,422 (6.9)
Service	2,057 (2.4)	737 (2.2)	1,320 (2.4)
Farmer	281 (0.3)	230 (0.6)	51 (0.1)
Blue collar	1,747 (2.3)	1,386 (4.5)	361 (0.7)
**HALex score, mean (range)**	0.74 (0.74–0.74)	0.75 (0.75–0.75)	0.73 (0.72–0.73)
**Age, mean (range) y**	74.6 (74.5–74.7)	74 (73.9–74.1)	75.1 (75–75.1)
**Comorbidities, mean (range), n**	1.40 (1.39–1.41)	1.48 (1.47–1.50)	1.34 (1.33–1.35)

Abbreviations: HALex, Health and Activities Limitation Index; NHIS, National Health Interview Survey.

a All values are frequency (weighted percentage), unless otherwise indicated.

Table 2 shows multivariable logistic regression results of modeling poor/fair self-rated health and lowest HALex quintile. Table 3 shows results of modeling multimorbidity stratified by sex (*P* < .001 for sex–occupation interaction) and modeling multiple functional limitations stratified by age subgroup (*P* = .006 for age–occupation interaction). After controlling for all other factors, unemployment had the highest odds of fair/poor health (Table 2), lowest HALex quintile (Table 2), and multiple functional limitations (Table 3), and among the highest odds of multimorbidity (Table 3). Service workers, farm workers, and blue collar workers did not differ from white collar workers in their likelihood of reporting fair/poor health and being in the lowest HALex quintile. Blue collar workers were significantly less likely than white collar workers to report multimorbidity (odds ratio [OR], 0.84; 95% confidence interval [CI], 0.74–0.97) or multiple functional limitations (OR, 0.84; 95% CI, 0.72–0.98). Male blue collar workers were at lower risk of multimorbidity than male white collar workers (OR, 0.75; 95% CI, 0.64–0.89); we did not find this difference among women.

**Table 2:** Multiple Logistic Regression Results for Fair/Poor Health and Lowest HALex Quintile, Participants Aged 65 or Older, National Health Interview Survey, 1997–2011

Health Indicator	Fair/Poor Health, Odds Ratio (95% CI)	Lowest HALex Quintile, Odds Ratio (95% CI)
**Education**		
High school or equivalent	0.60 (0.58–0.63)	0.63 (0.60–0.67)
More than high school	0.44 (0.42–0.47)	0.55 (0.51–0.58)
Less than high school	1 [Reference]	1 [Reference]
**Race/ethnicity**		
Hispanic	1.62 (1.52–1.73)	1.21 (1.13–1.30)
Black	1.61 (1.52–1.72)	1.38 (1.29–1.48)
Other	1.34 (1.22–1.48)	1.09 (0.96–1.24)
Non-Hispanic white	1 [Reference]	1 [Reference]
**Male**	1.18 (1.13–1.24)	0.98 (0.93–1.04)
**Age**	1.02 (1.02–1.02)	1.05 (1.05–1.06)
**Smoking**		
Former	1.35 (1.30–1.42)	1.38 (1.31–1.46)
Current	1.67 (1.56–1.79)	1.75 (1.62–1.90)
Never	1 [Reference]	1 [Reference]
**Alcohol consumption**		
Former	1.02 (0.97–1.08)	1.11 (1.05–1.18)
Current light	0.47 (0.45–0.50)	0.46 (0.43–0.49)
Current heavy	0.43 (0.38–0.48)	0.40 (0.34–0.46)
Never	1 [Reference]	1 [Reference]
**Employment/occupation**		
Unemployed	2.75 (2.46–3.07)	5.92 (4.82–7.26)
Service worker	1.10 (0.89–1.35)	0.83 (0.58–1.18)
Farm worker	0.86 (0.57–1.31)	1.04 (0.47–2.29)
Blue collar worker	1.16 (0.95–1.40)	0.91 (0.62–1.34)
White collar	1 [Reference]	1 [Reference]
**Weight status**		
Underweight	1.78 (1.59–2.00)	2.02 (1.80–2.27)
Overweight	1.04 (0.99–1.09)	0.91 (0.86–0.96)
Obese	1.61 (1.53–1.70)	1.56 (1.47–1.66)
Normal weight	1 [Reference]	1 [Reference]
**Marital status**		
Living with partner	1.18 (0.96–1.44)	1.16 (0.91–1.47)
Never married	0.94 (0.86–1.03)	1.41 (1.27–1.56)
Widowed	0.96 (0.92–1.01)	1.41 (1.33–1.50)
Divorced/separated	1.23 (1.15–1.32)	1.76 (1.63–1.89)
Married	1 [Reference]	1 [Reference]
**Survey year**	1.00 (0.99–1.00)	1.00 (1.00–1.01)

Abbreviations: CI, confidence interval; HALex, Health and Activities Limitation Index.

**Table 3:** Multiple Logistic Regression Results for Multiple Chronic Health Conditions, by Sex, and Multiple Functional Limitations, by Age Subgroup, Participants Aged 65 or Older, National Health Interview Survey, 1997–2011^a^

Health Indicator	Multiple Chronic Conditions			Multiple Functional Limitations		
	Overall	Men	Women	Overall	Young–Old (Aged 65–75)	Old–Old (Aged ≥76)
**Education**						
High school	0.84 (0.8–0.88)	0.91 (0.85–0.98)	0.80 (0.76–0.84)	0.73 (0.70–0.76)	0.71 (0.66–0.76)	0.76 (0.71–0.81)
>High school	0.85 (0.82–0.89)	0.95 (0.89–1.02)	0.79 (0.74–0.83)	0.66 (0.63–0.69)	0.62 (0.58–0.66)	0.71 (0.67–0.76)
<High school	1 [Ref]	1 [Ref]	1 [Ref]	1 [Ref]	1 [Ref]	1 [Ref]
**Race/ethnicity**						
Hispanic	0.78 (0.73–0.83)	0.68 (0.61–0.75)	0.86 (0.79–0.94)	0.82 (0.77–0.88)	0.79 (0.73–0.86)	0.88 (0.79–0.98)
Black	1.06 (1.01–1.12)	1.02 (0.94–1.11)	1.08 (1.01–1.16)	0.97 (0.91–1.03)	0.95 (0.88–1.03)	1.00 (0.92–1.09)
Other	0.96 (0.87–1.06)	0.85 (0.74–0.98)	1.03 (0.90–1.18)	0.95 (0.86–1.04)	0.97 (0.85–1.10)	0.93 (0.79–1.08)
Non-Hispanic white	1 [Ref]	1 [Ref]	1 [Ref]	1 [Ref]	1 [Ref]	1 [Ref]
**Male**	1.30 (1.25–1.35)	—	—	0.63 (0.60–0.65)	0.61 (0.57–0.64)	0.65 (0.61–0.69)
**Age**	1.02 (1.02–1.02)	1.02 (1.02–1.03)	1.02 (1.02–1.02)	1.07 (1.06–1.07)	1.05 (1.04–1.06)	1.09 (1.08–1.09)
**Smoking status**						
Former	1.61 (1.55–1.67)	1.65 (1.55–1.76)	1.59 (1.51–1.67)	1.28 (1.23–1.33)	1.29 (1.22–1.36)	1.28 (1.20–1.36)
Current	1.36 (1.28–1.44)	1.44 (1.31–1.58)	1.32 (1.22–1.43)	1.60 (1.51–1.70)	1.67 (1.55–1.79)	1.4 (1.24–1.57)
Never	1 [Ref]	1 [Ref]	1 [Ref]	1 [Ref]	1 [Ref]	1 [Ref]
**Alcohol consumption**						
Former	1.22 (1.16–1.27)	1.43 (1.32–1.54)	1.18 (1.11–1.25)	1.27 (1.21–1.33)	1.29 (1.20–1.38)	1.24 (1.16–1.33)
Current light	0.74 (0.71–0.78)	0.90 (0.83–0.98)	0.68 (0.65–0.72)	0.76 (0.73–0.79)	0.77 (0.72–0.82)	0.75 (0.70–0.80)
Current heavy	0.68 (0.62–0.75)	0.78 (0.70–0.88)	0.67 (0.56–0.82)	0.87 (0.80–0.95)	0.92 (0.83–1.02)	0.75 (0.64–0.89)
Never	1 [Ref]	1 [Ref]	1 [Ref]	1 [Ref]	1 [Ref]	1 [Ref]
**Employment/occupation**						
Unemployed	1.49 (1.39–1.60)	1.37 (1.23–1.51)	1.70 (1.54–1.87)	1.88 (1.75–2.02)	1.87 (1.72–2.03)	2.02 (1.72–2.36)
Service worker	1.09 (0.96–1.24)	1.19 (0.97–1.46)	1.08 (0.91–1.28)	0.82 (0.71–0.95)	0.84 (0.72–0.98)	0.74 (0.55–1.01)
Farm worker	0.92 (0.67–1.27)	0.88 (0.61–1.27)	1.16 (0.57–2.35)	1.04 (0.76–1.42)	0.91 (0.63–1.34)	1.40 (0.86–2.30)
Blue collar worker	0.84 (0.74–0.97)	0.75 (0.64–0.89)	1.13 (0.86–1.50)	0.84 (0.72–0.98)	0.87 (0.73–1.03)	0.73 (0.50–1.05)
White collar	1 [Ref]	1 [Ref]	1 [Ref]	1 [Ref]	1 [Ref]	1 [Ref]
**Obesity status**						
Underweight	1.11 (1.00–1.23)	1.36 (1.07–1.71)	1.04 (0.93–1.18)	1.38 (1.22–1.55)	1.61 (1.34–1.94)	1.27 (1.09–1.46)
Overweight	1.32 (1.28–1.37)	1.23 (1.16–1.30)	1.38 (1.31–1.45)	1.31 (1.25–1.36)	1.26 (1.20–1.34)	1.38 (1.30–1.46)
Obese	2.11 (2.02–2.21)	1.97 (1.83–2.12)	2.17 (2.04–2.30)	2.77 (2.64–2.91)	2.77 (2.60–2.95)	2.75 (2.54–2.98)
Normal weight	1 [Ref]	1 [Ref]	1 [Ref]	1 [Ref]	1 [Ref]	1 [Ref]
**Marital status**						
Never married	0.79 (0.73–0.86)	0.75 (0.67–0.84)	0.84 (0.75–0.93)	1.11 (1.02–1.20)	1.15 (1.04–1.28)	1.02 (0.91–1.15)
Living with partner	0.99 (0.84–1.17)	1.07 (0.87–1.32)	0.87 (0.67–1.12)	1.16 (0.98–1.37)	1.12 (0.92–1.37)	1.20 (0.87–1.67)
Widowed	1.03 (0.99–1.07)	0.90 (0.83–0.97)	1.08 (1.03–1.14)	1.11 (1.06–1.15)	1.15 (1.09–1.23)	1.06 (1.00–1.12)
Divorced/ separated	1.08 (1.02–1.14)	1.00 (0.92–1.08)	1.15 (1.07–1.24)	1.25 (1.18–1.33)	1.32 (1.23–1.41)	1.11 (0.99–1.23)
Married	1 [Ref]	1 [Ref]	1 [Ref]	1 [Ref]	1 [Ref]	1 [Ref]
**Survey year**	1.03 (1.03–1.04)	1.04 (1.03–1.04)	1.03 (1.03–1.04)	1.00 (1.00–1.01)	1.01 (1.00–1.01)	1.00 (0.99–1.00)

Abbreviation: Ref, reference.

a All values are odds ratios (95% confidence intervals).

Education, race/ethnicity, sex, age, health behaviors, obesity status, and marital status were all associated with health status measures. Non-Hispanic blacks reported poorer health than non-Hispanic whites on all outcomes except multiple functional limitations. Hispanics were at a higher risk than non-Hispanic whites for reporting fair/poor health and for being in the lowest HALex quintile; however, they were less likely to report multimorbidity and functional limitations. Men were more likely than women to report fair/poor health and multimorbidity; however, they were less likely to report multiple functional limitations. Having a high school education or more was associated with better health outcomes than having less than a high school education. Compared with never consuming alcohol, former alcohol consumption was associated with poorer health outcomes, while current alcohol consumption was associated with better health outcomes, even for heavy drinkers. Both former and current smoking was associated with poorer health across all outcomes when compared with never smoking. Both underweight and obese individuals were more likely to report fair/poor health than normal weight individuals. Being overweight was associated with a lower likelihood of being in the lowest HALex quintile but a higher likelihood of multimorbidity and functional limitations.

## Discussion

In this nationally representative sample of adults aged 65 or older, the typical older adult was a nonsmoker aged approximately 75 who had 1 or 2 chronic health conditions and did not work. Most employed adults worked in white collar occupations, only a small fraction worked in farming, and the rest were approximately equally distributed between blue collar and service occupations. Being unemployed/retired was associated with the greatest risk of poor health across all health status measures, even after controlling for smoking status, obesity, and other predictors of health. Blue collar and service workers had better outcomes than white collar workers on multimorbidity and functional limitations measures. Previous studies of middle-aged or working-age adults found white collar workers to have a lower likelihood of poor health (17,18), while a study of musculoskeletal disorders among shipyard employees found a higher prevalence of these disorders among white collar workers, possibly because blue collar workers with musculoskeletal problems transferred to white collar jobs (9). For older adults in more physically demanding occupations (such as service and blue collar) there might be a stronger healthy worker effect. As a result, healthier individuals are more likely to continue working, while those in poorer health are more likely to either exit the workforce or shift into less physically demanding white collar occupations (19). The lower likelihood of multiple chronic conditions and functional limitations among blue collar workers than among white collar workers might also reflect the benefit of greater lifetime physical activity in the workplace versus the mostly sedentary work of white collar occupations. We found a lower likelihood of multimorbidity among blue collar men but not blue collar women, which might indicate the differences in the types of blue collar jobs in which older adults of different sexes engage. In addition, for workers in jobs of lower socioeconomic status, employment can have stronger beneficial effect on health by increasing social support and income and by providing access to more comprehensive health insurance coverage (4,5,20).

Consistent with previous studies, sex, education, race/ethnicity, age, obesity status, drinking and smoking history, and marital status were associated with health outcome measures (7,21–23). Poorer outcomes across all measures were associated with having less than a high school education (compared with a high school education or more), with being underweight or obese (compared with normal weight), with being non-Hispanic black (compared with non-Hispanic white), and being divorced or separated (compared with being married). Former alcohol consumption was associated with a greater risk of all poor outcomes except fair/poor self-rated health, while current drinking was associated with better health outcomes, even for heavy drinkers. Alcohol abstinence in older adults was linked with loss of mobility and a higher risk of dementia, while low to moderate alcohol consumption was associated with lower overall mortality and higher HRQL, and heavy alcohol consumption was associated with higher bone mineral density (24,25). These data possibly indicate a genetic resiliency in older drinkers, with those most susceptible to the negative effects of alcohol either not surviving to older age or quitting drinking at a younger age.

Although most predictors resulted in consistent associations across outcomes, some had different effects depending on the outcome modeled. Hispanics were at a higher risk than non-Hispanic whites for having fair/poor health and being in the lowest HALex quintile; however, they were less likely to report multimorbidity and functional limitations. Poorer outcomes on health status measures incorporating self-perceived health in older Hispanics might be partially due to a difference in perception across cultures of what constitutes good health, as well as to limited access to health care in this group (26). Issues with access to care are also suggested by the lower risk of multimorbidity among Hispanics, possibly a result of chronic conditions being underdiagnosed in this group (26). However, limited access to care does not explain the lower risk of multiple functional limitations in this group that is consistent with previous frailty studies (27), and this discrepancy should be examined in future studies.

We also found that men were more likely than women to report fair/poor health and multimorbidity; however, men were less likely to report multiple functional limitations. That is, women were more likely to perceive their health as good or excellent and report fewer health conditions; however, they were more likely to have functional limitations. In previous studies, individuals in poor health did not necessarily report disability or limitations, and functional limitations were more likely to be reported if a person had limited availability and access to assistive devices (24). In addition, some of the observed differences might result from underreporting of functional limitations by men (24). Finally, the multimorbidity variable in our study did not include such disabling conditions as osteoporosis or resulting fractures, which are more common in women than men, possibly resulting in increased reports of limitations by women without increasing their risk of multimorbidity in our results (28).

This study used pooled cross-sectional data, and therefore causal inferences cannot be made. Past employment history information was not available, and although this study aimed to examine the effects of employment at older age, such effects may vary depending on whether the person re-entered the workforce after retirement and whether the person changed jobs or remained in career employment. Health-related inability to work was one component used to calculate HALex, and this component might have led to over-inflation of the association between employment and HALex, albeit to a small degree. Although previous NHIS data demonstrated a moderately high agreement between longest-held job and current job (29), this agreement might not apply to older workers, who are more likely to retire from their lifetime career employment and then seek new less demanding employment.

The major strength of this study was a large nationally representative data set obtained by pooling 15 years of data, with information on a range of sociodemographic and health status variables. The multivariable regression analysis used in this study allowed examination of the effects of multiple factors while controlling for the effects of potential confounders, and it was an improvement on previous studies reporting on prevalence. We also used 4 complementary health status measures to most comprehensively characterize the health of this population.

The prevalence of chronic conditions increases as people live longer with diseases; however, this does not necessarily translate into increased prevalence of functional limitations (30). These various aspects of a person’s health not only affect the person’s functioning differently but also require different amounts and kinds of health care resources to address different needs. In addition, as a growing number of older adults stay active in the workforce, occupational health resources need to be allocated differently to address the needs of the aging workforce. Older adults who continue working tend to be much healthier across multiple health outcomes, but perhaps providing better workplace accommodations for older adults with functional limitations would allow more of them to join the ranks of their healthier peers. Determination of the factors most closely associated with poor health outcomes across various health measures is therefore important for fine tuning the allocation of health care resources, both inside and outside the workplace, as the population ages.

In the current study, we characterized the health of the older US workers and nonworkers by examining the risk of 4 complementary poor health outcomes across various sociodemographic groups. We found a strong association between health status and employment/occupation and weaker associations for the following: education, race/ethnicity, sex, and smoking and drinking history. We also identified a variation in health status across different measures within population subgroups. Although some groups (eg, those with low levels of education, non-Hispanic blacks, the unemployed/retired) showed consistently poorer outcomes across all outcomes examined, the effects of some other predictors varied depending on the outcome. While these results bring to mind access to care issues (among Hispanics) as well as possibly lower availability and access to assistive devices (among women), they also suggest that poor health does not have to result in disability or poor quality of life. Future studies should examine the causes of such variation across outcomes and develop potential workplace intervention strategies for improving the health status of currently disadvantaged groups to enable them to remain in the workforce.

## References

[1] Statistics BoL. Labor force projections to 2022: the labor force participation rate continues to fall; 2013. http://www.bls.gov/opub/mlr/2013/article/labor-force-projections-to-2022-the-labor-force-participation-rate-continues-to-fall.htm. Accessed May 9, 2015.

[2] Bohle P , Pitts C , Quinlan M . Time to call it quits? The safety and health of older workers. Int J Health Serv 2010;40(1):23–41. 10.2190/HS.40.1.b 20198802

[3] Butler RN . The longevity revolution: the benefits and challenges of living a long life. New York (NY): Public Affairs; 2009.

[4] Fleming LE , Lee DJ , Martinez AJ , Leblanc WG , McCollister KE , Bridges KC , The health behaviors of the older US worker. Am J Ind Med 2007;50(6):427–37. 10.1002/ajim.20468 17503458

[5] Christ SL , Lee DJ , Fleming LE , LeBlanc WG , Arheart KL , Chung-Bridges K , Employment and occupation effects on depressive symptoms in older Americans: does working past age 65 protect against depression? J Gerontol B Psychol Sci Soc Sci 2007;62(6):S399–403. 10.1093/geronb/62.6.S399 18079428

[6] Luo N , Johnson JA , Shaw JW , Feeny D , Coons SJ . Self-reported health status of the general adult U.S. population as assessed by the EQ-5D and Health Utilities Index. Med Care 2005;43(11):1078–86. 10.1097/01.mlr.0000182493.57090.c1 16224300

[7] Skarupski KA , de Leon CF , Bienias JL , Scherr PA , Zack MM , Moriarty DG , Black-white differences in health-related quality of life among older adults. Qual Life Res 2007;16(2):287–96. 10.1007/s11136-006-9115-y 17033898

[8] Ostchega Y , Harris TB , Hirsch R , Parsons VL , Kington R . The prevalence of functional limitations and disability in older persons in the US: data from the National Health and Nutrition Examination Survey III. J Am Geriatr Soc 2000;48(9):1132–5. 10.1111/j.1532-5415.2000.tb04791.x 10983915

[9] Alexopoulos EC , Tanagra D , Konstantinou E , Burdorf A . Musculoskeletal disorders in shipyard industry: prevalence, health care use, and absenteeism. BMC Musculoskelet Disord 2006;7(1):88. 10.1186/1471-2474-7-88 17125504 PMC1676002

[10] Zahran HS , Kobau R , Moriarty DG , Zack MM , Holt J , Donehoo R ; Centers for Disease Control and Prevention (CDC). Health-related quality of life surveillance — United States, 1993–2002. MMWR Surveill Summ 2005;54(4):1–35. 16251867

[11] Pleis JR , Ward BW , Lucas JW . Summary health statistics for U.S. adults: National Health Interview Survey, 2009. Vital Health Stat 10 2010;(249):1–207. 21905346

[12] Fortin M , Bravo G , Hudon C , Vanasse A , Lapointe L . Prevalence of multimorbidity among adults seen in family practice. Ann Fam Med 2005;3(3):223–8. 10.1370/afm.272 15928225 PMC1466875

[13] Botman SL , Jack SS . Combining National Health Interview Survey datasets: issues and approaches. Stat Med 1995;14(5-7):669–77. 10.1002/sim.4780140523 7792456

[14] Manor O , Matthews S , Power C . Dichotomous or categorical response? Analysing self-rated health and lifetime social class. Int J Epidemiol 2000;29(1):149–57. 10.1093/ije/29.1.149 10750617

[15] Fried LP , Ferrucci L , Darer J , Williamson JD , Anderson G . Untangling the concepts of disability, frailty, and comorbidity: implications for improved targeting and care. J Gerontol A Biol Sci Med Sci 2004;59(3):255–63. 10.1093/gerona/59.3.M255 15031310

[16] Livingston EH , Ko CY . Use of the health and activities limitation index as a measure of quality of life in obesity. Obes Res 2002;10(8):824–32. 10.1038/oby.2002.111 12181392

[17] Krieger N , Barbeau EM , Soobader MJ . Class matters: U.S. versus U.K. measures of occupational disparities in access to health services and health status in the 2000 U.S. National Health Interview Survey. Int J Health Serv 2005;35(2):213–36. 10.2190/JKRE-AH92-EDV8-VHYC 15932004

[18] Lahelma E , Martikainen P , Rahkonen O , Roos E , Saastamoinen P . Occupational class inequalities across key domains of health: results from the Helsinki Health Study. Eur J Public Health 2005;15(5):504–10. 10.1093/eurpub/cki022 16014660

[19] McMichael AJ . Standardized mortality ratios and the “healthy worker effect”: scratching beneath the surface. J Occup Med 1976;18(3):165–8. 10.1097/00043764-197603000-00009 1255276

[20] Blanc PD , Katz P , Yelin E . Mortality risk among elderly workers. Am J Ind Med 1994;26(4):543–7. 10.1002/ajim.4700260411 7810552

[21] Freedman VA , Martin LG , Schoeni RF . Recent trends in disability and functioning among older adults in the United States: a systematic review. JAMA 2002;288(24):3137–46. 10.1001/jama.288.24.3137 12495394

[22] Louie GH , Ward MM . Socioeconomic and ethnic differences in disease burden and disparities in physical function in older adults. Am J Public Health 2011;101(7):1322–9. 10.2105/AJPH.2010.199455 21164082 PMC3110229

[23] Rettenmaier AJ , Wang Z . What determines health: a causal analysis using county level data. Eur J Health Econ 2013;14(5):821–34. 10.1007/s10198-012-0429-0 22961232

[24] LaCroix AZ , Guralnik JM , Berkman LF , Wallace RB , Satterfield S . Maintaining mobility in late life. II. Smoking, alcohol consumption, physical activity, and body mass index. Am J Epidemiol 1993;137(8):858–69. 8484377 10.1093/oxfordjournals.aje.a116747

[25] Mukamal KJ , Kuller LH , Fitzpatrick AL , Longstreth WT Jr , Mittleman MA , Siscovick DS . Prospective study of alcohol consumption and risk of dementia in older adults. JAMA 2003;289(11):1405–13. 10.1001/jama.289.11.1405 12636463

[26] Shetterly SM , Baxter J , Mason LD , Hamman RF . Self-rated health among Hispanic vs non-Hispanic white adults: the San Luis Valley Health and Aging Study. Am J Public Health 1996;86(12):1798–801. 10.2105/AJPH.86.12.1798 9003141 PMC1380737

[27] Espinoza SE , Jung I , Hazuda H . Lower frailty incidence in older Mexican Americans than in older European Americans: the San Antonio Longitudinal Study of Aging. J Am Geriatr Soc 2010;58(11):2142–8. 10.1111/j.1532-5415.2010.03153.x 21054295 PMC3058917

[28] Murtagh KN , Hubert HB . Gender differences in physical disability among an elderly cohort. Am J Public Health 2004;94(8):1406–11. 10.2105/AJPH.94.8.1406 15284051 PMC1448463

[29] Gómez-Marín O , Fleming LE , Caban A , Leblanc WG , Lee DJ , Pitman T . Longest held job in U.S. occupational groups: the National Health Interview Survey. J Occup Environ Med 2005;47(1):79–90. 15643162 10.1097/01.jom.0000147213.76606.55

[30] Crimmins EM . Trends in the health of the elderly. Palo Alto (CA): Annual Reviews; 2004. 10.1146/annurev.publhealth.25.102802.124401 15015913

